# Map-based cloning and CRISPR/Cas9-based editing uncover *BoNA1* as the causal gene for the no-anthocyanin-accumulation phenotype in curly kale (*Brassica oleracea* var. *sabellica*)

**DOI:** 10.1093/hr/uhad133

**Published:** 2023-06-29

**Authors:** Kaiwen Yuan, Xinyu Zhao, Wenru Sun, Limei Yang, Yangyong Zhang, Yong Wang, Jialei Ji, Fengqing Han, Zhiyuan Fang, Honghao Lv

**Affiliations:** State Key Laboratory of Vegetable Biobreeding, Institute of Vegetables and Flowers, Chinese Academy of Agricultural Sciences, Beijing, 100081, China; State Key Laboratory of Vegetable Biobreeding, Institute of Vegetables and Flowers, Chinese Academy of Agricultural Sciences, Beijing, 100081, China; State Key Laboratory of Vegetable Biobreeding, Institute of Vegetables and Flowers, Chinese Academy of Agricultural Sciences, Beijing, 100081, China; State Key Laboratory of Vegetable Biobreeding, Institute of Vegetables and Flowers, Chinese Academy of Agricultural Sciences, Beijing, 100081, China; State Key Laboratory of Vegetable Biobreeding, Institute of Vegetables and Flowers, Chinese Academy of Agricultural Sciences, Beijing, 100081, China; State Key Laboratory of Vegetable Biobreeding, Institute of Vegetables and Flowers, Chinese Academy of Agricultural Sciences, Beijing, 100081, China; State Key Laboratory of Vegetable Biobreeding, Institute of Vegetables and Flowers, Chinese Academy of Agricultural Sciences, Beijing, 100081, China; State Key Laboratory of Vegetable Biobreeding, Institute of Vegetables and Flowers, Chinese Academy of Agricultural Sciences, Beijing, 100081, China; State Key Laboratory of Vegetable Biobreeding, Institute of Vegetables and Flowers, Chinese Academy of Agricultural Sciences, Beijing, 100081, China; State Key Laboratory of Vegetable Biobreeding, Institute of Vegetables and Flowers, Chinese Academy of Agricultural Sciences, Beijing, 100081, China

## Abstract

*Brassica oleracea* comprises several important vegetable and ornamental crops, including curly kale, ornamental kale, cabbage, broccoli, and others. The accumulation of anthocyanins, important secondary metabolites valuable to human health, in these plants varies widely and is responsible for their pink to dark purple colors. Some curly kale varieties lack anthocyanins, making these plants completely green. The genetic basis of this trait is still unknown. We crossed the curly kale inbred line BK2019 (without anthocyanins) with the cabbage inbred line YL1 (with anthocyanins) and the Chinese kale inbred line TO1000 (with anthocyanins) to generate segregating populations. The no-anthocyanin trait was genetically controlled by a recessive gene, *bona1*. We generated a linkage map and mapped *bona1* to a 256-kb interval on C09. We identified one candidate gene, *Bo9g058630*, in the target genomic region; this gene is homologous to *AT5G42800*, which encodes a dihydroflavonol-4-reductase-like (DFR-like) protein in *Arabidopsis*. In BK2019, a 1-bp insertion was observed in the second exon of *Bo9g058630* and directly produced a stop codon. To verify the candidate gene function, CRISPR/Cas9 gene editing technology was applied to knock out *Bo9g058630*. We generated three *bona1* mutants, two of which were completely green with no anthocyanins, confirming that *Bo9g058630* corresponds to *BoNA1.* Different insertion/deletion mutations in *BoNA1* exons were found in all six of the other no-anthocyanin kale varieties examined, supporting that independent disruption of *BoNA1* resulted in no-anthocyanin varieties of *B. oleracea*. This study improves the understanding of the regulation mechanism of anthocyanin accumulation in *B. oleracea* subspecies.

## Introduction

Anthocyanins are water-soluble pigments that constitute a group of natural secondary metabolites in plants [1, 2]. Anthocyanins protect plants against many environmental stresses, including ultraviolet radiation, low temperature, heat, drought, salinity, heavy metals, and pathogen infection [3, 4]. In addition, anthocyanins have antioxidant properties, making them functional compounds that reduce the incidence of chronic diseases to protect human health [5].

The anthocyanin biosynthetic pathway in *Arabidopsis thaliana* has been thoroughly studied [6]. Anthocyanin biosynthesis is catalyzed by several enzymes, such as phenylalanine ammonia lyase, chalcone synthase, dihydroflavonol 4-reductase (DFR), UDP-glucosyltransferase, and so on [7, 8]. These structural genes are regulated by transcription factors, such as R2R3-MYB transcription factors (MYB113 and MYB114), basic helix–loop–helix transcription factors (TT8), WD40, and several other transcription factors [9, 10].

The genus *Brassica* in the family *Brassicaceae* contains more than 37 species, including several commercially important plants [11, 12]. Among them, *Brassica oleracea* includes several important vegetable and ornamental members, including cabbage, cauliflower, broccoli, kohlrabi, curly kale, ornamental kale, and others [13]. *Brassica oleracea* plants are extremely diverse with respect to anthocyanin accumulation, as the color of these plants can range from green to dark purple. Although several studies have reported genomic loci/genes associated with anthocyanin accumulation in *B. oleracea* [14–16], the genetic basis of anthocyanin biosynthesis is still poorly understood. Anthocyanin biosynthesis-related genes throughout the whole *B. oleracea* genome have been identified through searches of syntenic and nonsyntenic orthologs against genes in *A. thaliana* [17, 18]. In purple cauliflower, an R2R3 MYB transcription factor was activated, resulting in anthocyanin accumulation [19]. This gene was also believed to be an important transcription factor in other purple *B. oleracea* subspecies [20]. Song et al. found that *BoMYBL2–1*, a negative regulator, was associated with the purple trait of red cabbage [21]. Several researchers finely mapped the genomic loci for anthocyanin accumulation in white/purple ornamental kale [16, 22, 23] and identified *BoDFR* as the candidate gene involved in the development of the pink leaf trait [16, 23]. While studying the purple ornamental kale variety Red Dove, Zhang et al. [24] found that anthocyanin biosynthesis could be induced with low temperature and that the positive regulators *BoPAP1, BoMYB113*, and *BoMYB114* were upregulated during this process. Additionally, other studies have shown that *BoLBD37L* and *BoTT8* may play roles in anthocyanin accumulation in cabbage and cauliflower [25, 26].

While most *B. oleracea* plants produce anthocyanins, a few curly kale varieties lack anthocyanins and thus have a completely green phenotype. In this research, we characterized a critical gene, *BoNA1*, which was connected with controlling anthocyanin biosynthesis in *B. oleracea*. Moreover, haplotype analysis of different accessions revealed that independent disruption of *BoNA1* resulted in no-anthocyanin kale varieties.

## Results

### Phenotypic and genetic analysis of the green/purple trait

**Figure 1:**
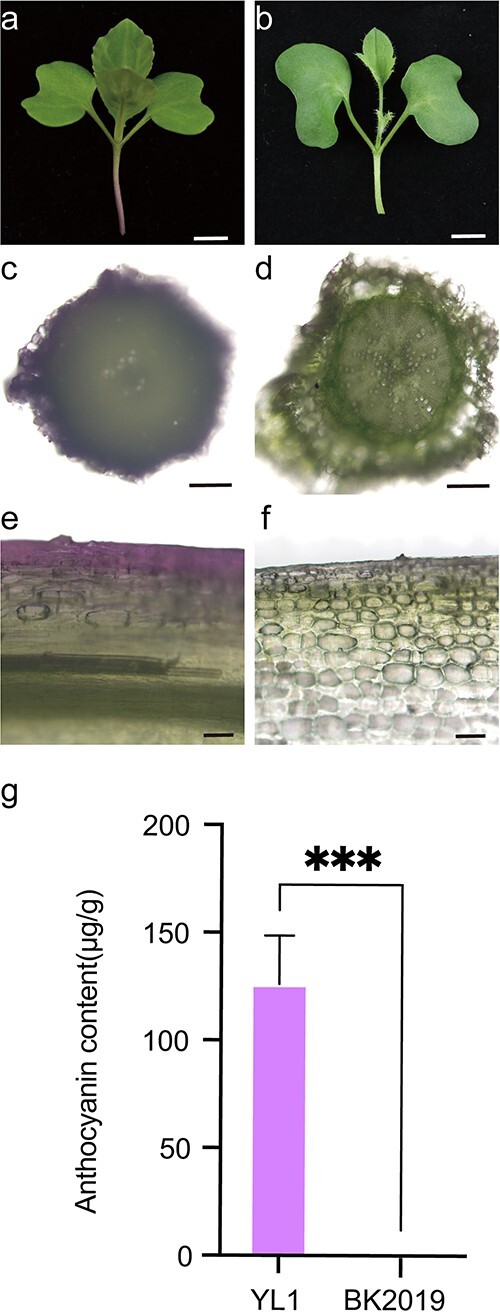
Phenotypic comparison of the green and purple traits of YL1 and BK2019. **(a)** Parental line with purple hypocotyls (YL1). Bar = 4 mm. **(b)** Parental line with green hypocotyls (BK2019). Bar = 4 mm. **(c)**, **(d)** Transverse sections of the hypocotyls of YL1 and BK2019. Bars = 0.2 mm. **(e)**, **(f)** Transverse sections of young leaves of YL1 and BK2019. Bars = 50 μm. **(g)** Total anthocyanin contents in the hypocotyls of YL1 and BK2019. The error bars indicate the standard deviations of three replicates. The asterisks represent significant differences according to T tests (^***^, p < 0.001).

In the cabbage inbred line YL1, anthocyanin pigments were found to accumulate and were clearly detected in the hypocotyls and young leaves of approximately 10-day-old seedlings (Fig. 1a). The curly kale inbred line BK2019 was completely green during the whole growth period, including in the hypocotyls and leaves of seedlings (Fig. 1b). Analysis of tissue cross-sections revealed that anthocyanins were found mainly in the veins of young leaves and the epidermal cells of hypocotyls of YL1 (Fig. 1c, e). However, in the hypocotyls and leaves of BK2019, no purple color was observed (Fig. 1d, f). To confirm the presence/absence of anthocyanins, we measured the total anthocyanins in the hypocotyls of the 10-day-old seedlings of these two lines. The results indicated that the anthocyanin levels in YL1 and BK2019 were 126 and 0 μg/g, respectively (Fig. 1g).

BK2019 was crossed with YL1 to generate F_1_ and F_2_ progeny. All 24 F_1_ individuals had purple hypocotyls. A total of 2020 individuals were obtained in the F_2_ population, of which 1512 individuals displayed purple hypocotyls, while 508 were green. A chi-square test verified this 3:1 segregation ratio (χ^2^ = 0.0238 < χ^2^_0.05,1_ = 3.84; *P* = 0.8775) (Table 1). In addition, another F_2_ population derived from TO1000 (Chinese kale, with anthocyanins) × BK2019 reflected the same segregation ratio (Table 1). Based on these results, we confirmed that a single recessive gene, which we named *Bona1*, controlled the no-anthocyanin trait.

**Table 1:** Genetic analyses of the no-anthocyanin trait of BK2019 in different segregating populations

Cross parents	Population names	Total	Purple plants	Green plants	Ratio	χ^2^	P value
-	P1 (YL1)	15	15	0	-	-	-
-	P2 (TO1000)	15	15	0	-	-	
-	P3 (BK2019)	15	0	15	-	-	-
P1 × P3	F1	25	25	0	-	-	-
	F2	2020	1512	508	3:1	0.0238	0.878
P2 × P3	F1	25	25	0	-	-	-
	F2	1051	789	262	3:1	0.00285	0.957

χ^2^ < χ^0.05^ (3.84) was considered significant.

### Map-based cloning of the gene controlling the no-anthocyanin trait of BK2019

**Figure 2:**
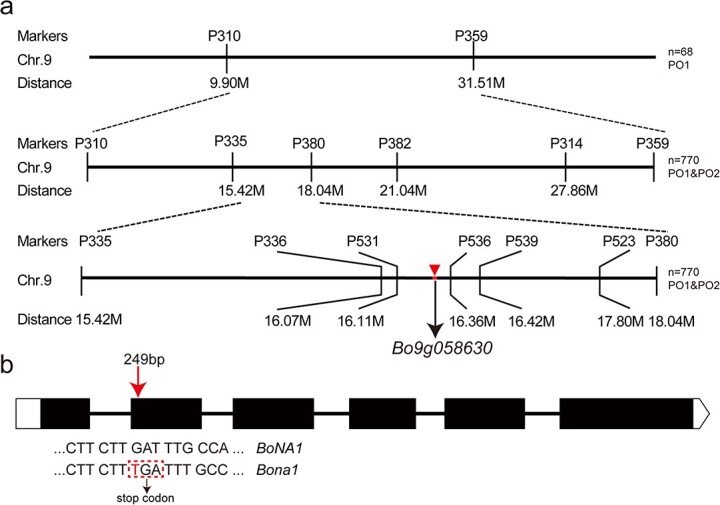
Map-based cloning of *BoNA1.***(a)** Fine mapping of *BoNA1* with InDel markers. The triangle indicates the location of candidate gene. **(b)** Schematic diagram depicting the exons (solid boxes) and introns (straight lines) of *BoNA1*. The arrow indicates the position of the *bona1* mutation.

We sequenced YL1 and BK2019 to identify the nucleotide variations between the parent lines. Based on their insertion and deletion (InDel) variations, we designed 98 primers whose targets were evenly distributed across the nine chromosomes and screened for polymorphisms between YL1 and BK2019, as well as the two DNA pools (the Pur pool comprised 10 purple F_2_ individuals, and the Gre pool comprised 10 green F_2_ individuals). Two polymorphic markers, namely, P310 (C09: 9904621) and P359 (C09: 31514900), were identified on C09. Using 48 F_2_ individuals (24 purple individuals and 24 green individuals) that were randomly selected, we confirmed that P310 and P359 were flanking and linked to the no-anthocyanin trait, and we found that the genetic distances of P310 and P359 were 8.5 and 22.8 cM, respectively (Fig. 2a).

To further narrow the target region, we developed nine polymorphic markers to genotype the individuals of the two F_2_ populations. Although all 3071 individuals were genotyped, only green individuals were used for genetic mapping and for constructing the linkage map (Fig. 2a). Ultimately, *Bona1* was mapped to a 256-kb (C09: 16, 889, 226 … 17, 151, 416) region flanked by the InDel markers P531 and P536; the genetic distances were 0.01 and 0.02 cM, respectively (Fig. 2a).

### *Bo9g058630* is a strong candidate gene for *BoNA1*

Based on the annotations on the TO1000 reference genome website [27], 35 genes are located in the 256-kb region. The results of a BLAST analysis and functional annotations suggested that only a structural gene, *Bo9g058630*, which encodes a dihydroflavonol-4-reductase-like protein homologous to Arabidopsis AT5G42800, is related to anthocyanin biosynthesis. Therefore, we hypothesized that the gene, *Bo9g058630,* is responsible for the no-anthocyanin trait of BK2019.

We designed the primer pair *BoNA1-F/R* to amplify the gene in YL1 and BK2019. Sequencing analysis showed that *Bo9g058630* has a total length of 1580 bp in YL1 and that the gene comprises six exons and five introns (Fig. 2b). A 1-bp nucleotide insertion (T at position 249 downstream of the initiator codon) in the second exon was identified in BK2019, which directly resulted in a premature stop codon (TGA), and 336 amino acids in the C-terminus were truncated in the mutant allele compared with the normal allele in YL1 (Fig. 2b). Therefore, we hypothesized that *Bo9g058630* was the causal gene responsible for the no-anthocyanin trait of BK2019.

### Functional verification of *BoNA1*

**Figure 3:**
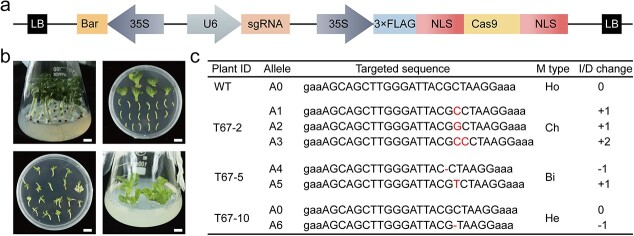
Functional verification of *BoNA1* based on the results of the CRISPR/Cas9 system. **(a)** Structure of the CRISPR/Cas9-BoNA1 vector for transformation. Cas9 can be used to identify transgenes driven by the 35S promoter, and sgRNA is driven by the U6 promoter. **(b)** Different stages of cabbage transformation. **(c)** Mutations at the target region of *BoNA1* in the T0 transgenic lines. Plant ID represents the plant ID number, in which exon 4 of *BoNA1* was targeted, except in the wild type (WT). The letters/dashes (“-”) in red indicate nucleotide changes/deletions. Ho, Ch, Bi and He represent homozygous, chimeric mutation, biallelic mutation, and heterozygous mutation, respectively. I/D changes indicate inserted/deleted nucleotides, “+”/“-” stands for insertions/deletions, respectively.

To confirm the function of *BoNA1*, we knocked out *BoNA1* in YL1 cells via the CRISPR/Cas9 genome-editing system. A *CRISPR/Cas9-BoNA1* vector with one sgRNA targeting the fourth exon of *BoNA1* was subsequently constructed (Fig. 3a). According to the established methods of *Agrobacterium*-mediated transformation in cabbage [28], approximately 1000 explants were used for transformation, and we ultimately obtained 17 Basta-resistant plants (Fig. 3b). To verify the presence of the transgene in these lines, the primers *Cas9-F/R* and *Bar-F/R,* which were designed based on the *Cas9* gene sequence and resistance gene (Basta) sequence, were used to detect all the Basta-resistant plants. The results showed that 8 of the 17 lines were positive transgenic plants.

To identify the mutations in *BoNA1* in the eight T0 transgenic plants, the *BoNA1-F/R* primers were used to amplify the *BoNA1* gene, after which the amplicons were Sanger-sequenced. Finally, 3 (T67-2, T67-5, T67-10) of the eight T0 transgenic plants were found to harbor mutations in the targeted region (Supplementary Fig. S1), corresponding to 37.5% gene editing efficiency.

To determine the mutation types, we cloned the amplicons of the *BoNA1-F/R* primers into a T-vector and sequenced at least 20 independent clones per plant. The sequences around the target region in these two lines are shown in Fig. 3c. In T67-2, there were three mutations, namely, two 1 bp insertions (C, 7/20; G, 9/20) and one 2-bp insertion (CC, 4/20), suggesting that the mutation was chimeric. In T67-5, there were two mutations, including a 1-bp deletion (13/20) and a 1-bp insertion (7/20), suggesting that the mutation was biallelic. Only one mutation (1 bp deletion, 8/20) was found in T67-10, suggesting that the mutation was heterozygous.

**Figure 4:**
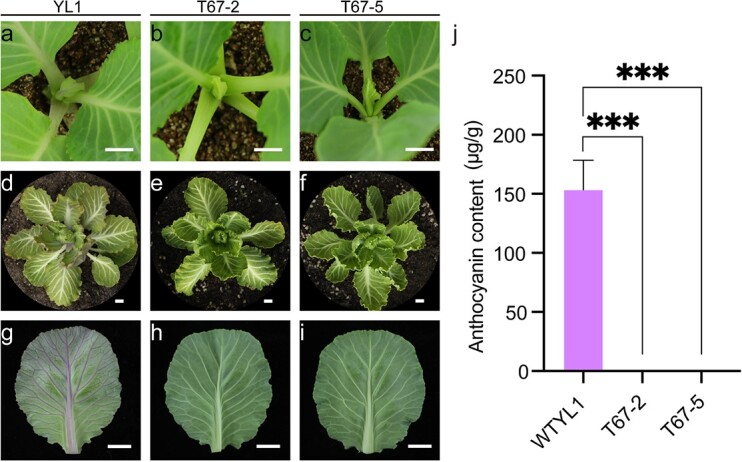
Phenotypes resulting from the YL1 and *bona1* mutations. **(a)**-**(c)** Phenotypes of YL1, T67–2, and T67–5 grown under 25°C and an 18-hour light/6-hour dark photoperiod. Bars = 5 mm. **(d)**- **(i)** Phenotypes of YL1, T67-2, and T67-5 that were grown under 4°C and an 18-hour light/6-hour dark photoperiod. Bars = 1 cm. **(j)** Anthocyanin levels in YL1, T67-2, and T67-5. The error bars indicate the standard deviations of three replicates. The asterisks represent significant differences according to T tests (^***^, *p* < 0.001).

The *bona1* mutants and YL1 were first transplanted into soil and then cultivated in a normal growth chamber. Among the three *bona1* mutants, T67-2 and T67-5 had green leaves, in contrast to YL1, which had obvious purple young leaves and purple margins on mature leaves (Fig. 4a–c). As low temperature induces the accumulation of anthocyanins [29], enabling a better visualization of purple/green phenotypes, we further grew these plants at 4°C. The leaves, stems and veins of YL1 were obviously purple, whereas those of the two *bona1* mutants were still green (Fig. 4d–i). The total anthocyanins in the stems of the 4°C-treated plants were then extracted and measured. The anthocyanin content in YL1 was 153 μg/g, while that in the two mutants was 0 μg/g (Fig. 4j). These results indicated that in *B. oleracea BoNA1* is essential for anthocyanin biosynthesis and loss of *BoNA1* function leads to a no-anthocyanin trait*.*

### Phylogenetic tree of BoNA1

**Figure 5:**
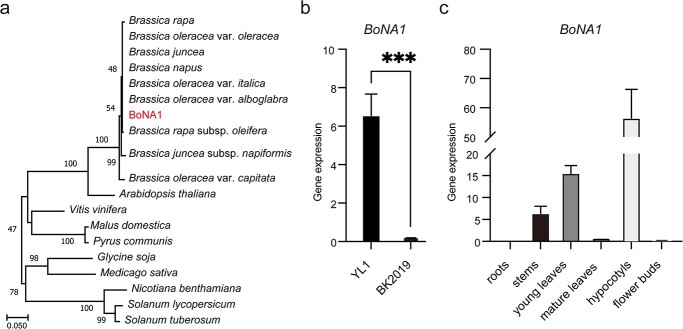
Gene expression patterns of *BoNA1.***(a)** Phylogenetic analysis of BoNA1 and its related proteins. **(b)** Expression levels of *BoNA1* in the hypocotyls of YL1 and BK2019. **(c)** qRT–PCR analysis results of *BoNA1* expression patterns in different tissues of YL1. The error bars indicate the standard deviations of three replicates. The asterisks represent significant differences according to *t* tests (^***^, p < 0.001).

To study the phylogenetic relationships between BoNA1 and its homologous proteins in other species, we searched the National Center for Biotechnology Information (NCBI) protein database using BLASTP with the BoNA1 sequence as a query. We then downloaded the sequences of 18 homologous proteins and constructed a phylogenetic tree (Fig. 5a). Our results revealed that BoNA1 was highly conserved in the *Brassica* family, suggesting that the function of BoNA1 as a DFR in the *Brassica* family was conserved.

### Tissue expression patterns of *BoNA1*

To analyze the transcript accumulation of *BoNA1,* quantitative real-time PCR (qRT-PCR) was applied. In the seedling stage, the expression of *BoNA1* in YL1 was much higher than that in BK2019 (Fig. 5b). In YL1, *BoNA1* was highly expressed in hypocotyls, stems, and young leaves; moderately expressed in flower buds and mature leaves; and not expressed in roots (Fig. 5c). These results were in accordance with the shade of purple color of these tissues, which further indicated that *BoNA1* is closely related to anthocyanin biosynthesis.

### Independent disruption of *BoNA1* results in no-anthocyanin varieties of *B. oleracea*

**Figure 6:**
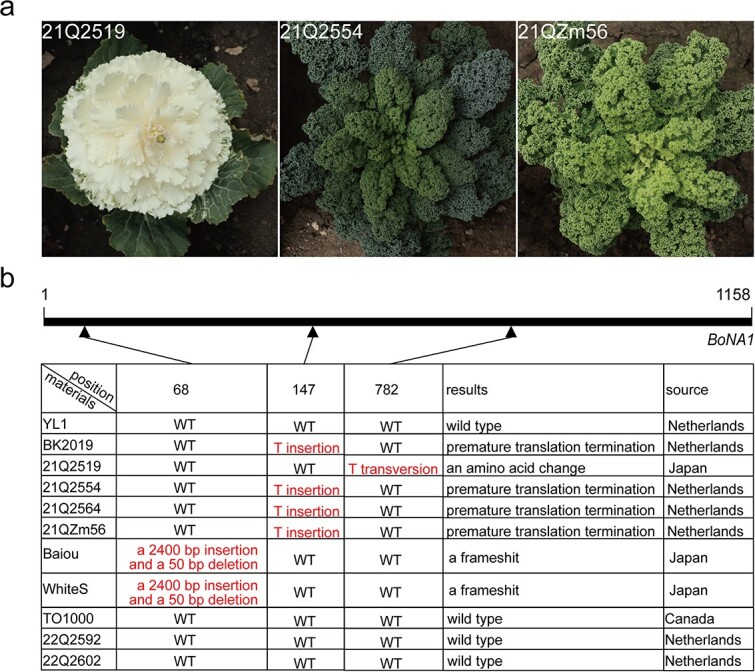
Phenotypes and sequence analysis of *BoNA1* in no-anthocyanin/anthocyanin-containing *B. oleracea*. **(a)** Phenotypes of 21Q2554, 21QZm56, and 21Q2519. **(b)** Sequence analysis of the *BoNA1* coding sequences in YL1; BK2019; six kale varieties, namely, 21Q2519, 21Q2554, 21Q2564, 21QZm56, White S, and Baiou; and three anthocyanin accumulation materials, namely TO1000, 22Q2592, and 22Q2602. YL1 was used for the wild-type sequence of *BoNA1*. The triangles indicate the loci of the mutations. Red indicates changed bases.

We selected several other curly kale and ornamental kale varieties that lack or produce anthocyanins to test whether they harbor mutations in the *BoNA1* gene (Fig. 6a, Supplementary Fig. S2). The primers *BoNA1-F/R* successfully amplified and sequenced *BoNA1* in the 11 materials. Multiple sequence alignment results suggested that the coding DNA sequence (CDS) of *BoNA1* in the seven materials lacking anthocyanins (BK2019, 21Q2519, 21Q2554, 21Q2564, 21QZm56, Baiou, and White S) had several mutations and that the three materials with anthocyanins (TO1000, 22Q2592, and 22Q2602) had no mutations (Fig. 6b). 21QZm56, 21Q2554, and 21Q2564 had the same mutation, consistent with that in BK2019. In White S and Baiou ornamental kale, an insertion and a deletion were identified in exon 1 of *BoNA1*, which resulted in a frameshift. In 21Q2519, two single-nucleotide polymorphisms (SNPs) were discovered in the CDS of *BoNA1*, one of which resulted in a nonsynonymous mutation (782T to A) that resulted in an amino acid change from Leu to His. These results suggested that no-anthocyanin varieties of *B. oleracea* arose from independent disruptions of *BoNA1.*

### Different expression level of *BoNA1* is correlated with varying degrees of purple coloration in *B. oleracea* vegetables

The accumulation of anthocyanins varies widely and is responsible for the pink to dark purple colors in *B. oleracea*. Compared with YL1, 22Q2592 and 22Q2602 had no mutations in the exons of *BoNA1*. We further investigated whether mutations in the promoter of *BoNA1* affected the expression of *BoNA1* and resulted in phenotypes with varying degrees of purple color in these three materials. We first sequenced the promoter of *BoNA1* in these three materials and found three InDels and several SNPs. Using PlantCARE (http://bioinformatics.psb.ugent.be/webtools/plantcare/html/), we found only an AE-box (AGAAACTT) related to the light response in *A. thaliana* among the InDels (Supplementary Fig. S3). Furthermore, the expression of *BoNA1* was measured in these three materials (Supplementary Fig. S4). Interestingly, among the three materials, the *BoNA1* expression level was positively correlated with the anthocyanin level. These results suggest that mutation of the promoter of *BoNA1* affects the expression of *BoNA1* and results in varying degrees of purple coloration in *B. oleracea* vegetables.

## Discussion

Due to increasing emphasis on improving living standards, increasing attention has been given to the health-promoting function of anthocyanins [30]. *B. oleracea* vegetables contain anthocyanins and thus have great value to the human diet. Moreover, differences in the accumulation of anthocyanins result in *B. oleracea* with attractive colors, thus, some cultivars are planted during the cool season for ornamental purposes. To study the genetic basis of the no-anthocyanin trait of BK2019, a map-based cloning strategy was used to clone *BoNA1*, a recessive gene that is closely related to anthocyanin synthesis. CRISPR/Cas9 gene editing and *Agrobacterium*-mediated transformation showed that *bona1* mutants contained no anthocyanins, and we ultimately verified the function of this gene. Given our findings, we conclude that *BoNA1* is required for anthocyanin biosynthesis in *B. oleracea*.

### *BoNA1* is an essential structural gene for the accumulation of anthocyanins in kale

Although the genetic regulation of anthocyanins has already been well studied in the model plant species *A. thaliana* [6, 7], few genes have been successfully cloned in *B. oleracea*. In *Arabidopsis,* DFR has been recognized as an important enzyme in the mid-to-late stages of the anthocyanin biosynthetic pathway [31]. In several *B. oleracea* subspecies, including ornamental kale [14, 16, 23], Chinese kale (*Brassica oleracea var. alboglabra*) [15] and cabbage (*Brassica oleracea var. capitata f. alba*) [32], a link between *BoDFR* and the accumulation of anthocyanins has been hypothesized. Sequencing analysis showed different mutations in the CDS of *BoDFR* in these materials, which requires further functional verification. In this research, we used a curly kale variety, BK2019, which is completely green, lacks anthocyanins, and has not been studied before, to carry out map-based cloning of *bona1*. Our results suggest that in the non–anthocyanin-accumulating kale varieties, there are three types of *BoNA1*, namely, one type with a 1-bp insertion that leads to a stop codon, one type with one synonymous mutation and one nonsynonymous mutation (782T to A), and one type with a 2400-bp insertion and a 50-bp deletion in exon 1. Our findings covered nearly all of the previous research results on *BoNA1* variants. Thus, we suspect that *BoNA1* has various mutations, which may explain why non–anthocyanin-accumulating kale varieties do not contain any anthocyanins, and that *BoNA1* experienced an independent loss of function during the process of artificial domestication.

*Brassica oleracea* vegetables has varying degrees of anthocyanin accumulation and then, their colors range from pink to dark purple. Although 22Q2592, 22Q2602, and YL1 had the same CDS sequence of *BoNA1,* there was a significant difference in their colors. We sequenced the promoter sequence of *BoNA1* in these three materials and found that 22Q2592 and 22Q2602 had the same sequences but were not consistent with YL1. We suspected that these different sequences were correlated with the expression of *BoNA1* and thus the colors between YL1 and the other two materials were completely different. As for 22Q2592 and 22Q2602, a different expression of *BoNA1* was also found, although the promoter sequence of *BoNA1* was consistent, further studies are needed to determine whether this result was correlated to the expression of transcription factors that was related to anthocyanin biosynthesis.

### CRISPR/Cas9 editing is an efficient technique for gene functional verification

In recent years, three genome editing techniques have enabled the direct targeting of regions of genes in a DNA sequence-specific manner, and CRISPR/Cas9 gene editing, which is a third-generation genome editing technique, and its predecessors that involved zinc-finger nucleases and transcription activator-like effector nucleases [33, 34]. CRISPR/Cas9 gene editing can edit targets specifically, leading to loss of gene function and has great application prospects in various fields of biology [35, 36]. To date, CRISPR/Cas9 gene editing has been successfully applied in various plant species, including rice (*Oryza sativa* L.) [37], *A. thaliana* [38], maize (*Zea mays* L.) [39], and others. *B. oleracea*, *Brassica napus*, and other *Brassicaceae* plants [40, 41] have also been successfully edited with the CRISPR/Cas9 system. In this research, we successfully used this system to edit *BoNA1* in cabbage YL1 and ultimately obtained three *bona1* mutants. No homozygous mutations were found in these three lines, and two mutants showed a non–anthocyanin-accumulating phenotype, which was consistent with the phenotype of BK2019.

Three transgenic plants from the eight T0 transgenic plants, or approximately 38%, were identified as *bona1* mutants. In a study on Chinese kale in which one sgRNA sequence targeted two genes (*BaPDS1* and *BaPDS2*), approximately 76.47% of the transgenic plants harbored mutations [40]. In a study on *B. napus* in which four sgRNA sequences targeted *BnaA5_SBE2.1, BnaC4_SBE2.1 BnaA10_SBE2.2*, and *BnaAnn_SBE2.1*, 29 transgenic plants among 36 positive T0-independent lines, approximately 80.5%, were identified as mutants [41].

## Materials and methods

### Plant material

YL1 is an inbred line of cabbage. Its leaves are yellow-green, but the hypocotyls are purple. TO1000 is a Chinese kale line with dark green leaves and purple hypocotyls. BK2019 is a curly kale inbred line, and both its leaves and hypocotyls are green. YL1 and TO1000 were used as female parents. These two inbred lines were crossed with BK2019, which was used as the male parent, to generate F_1_ plants. After that, F_2_ populations that were used for mapping *BoNA1* were obtained from self-pollination of F_1_ plants.

### Anthocyanin observations, extractions and measurements

YL1 and TO1000 contained small amounts of anthocyanins, but BK2019 did not contain any anthocyanins. The parents, F_1_ plants and F_2_ plants could be clearly distinguished by observing the hypocotyls and young leaves of 10- to 15-day-old seedlings with the naked eye. Freehand sectioning was used to obtain transverse sections of hypocotyls and young leaves of YL1 and BK2019. The distribution of anthocyanins in the hypocotyls and young leaves was observed and imaged with an optical microscope (Olympus, Japan).

The hypocotyls of YL1 and BK2019 were used to measure the total anthocyanin content. The hypocotyls (0.1 g) of 10- to 15-day-old seedlings were first milled to a powder after flash-freezing and then extracted in 2 ml of 80% ethanol (pH of 1.5) for 4 h at 40°C in the dark. The solutions were then centrifuged at 12 000 rpm for 10 min in a centrifuge that had been precooled to 4°C. After that, the obtained supernatant was transferred to a new tube, after which its absorption at 530, 620, and 650 nm was measured using an EV-2200 spectrophotometer (Onlab, Shanghai, China). The following formula was used to compute the anthocyanin level: OD_λ_/(4.62 × 10^4^) × V/m × 10 [6], where OD_λ_ = (OD_530_ − OD_620_) − 0.1(OD_650_ − OD_620_).

### DNA extraction, whole-genome sequencing, and primer design

Using the cetyltrimethyl ammonium bromide (CTAB) method [42], total DNA was extracted from the plants composing the two F2 populations, the three parental lines (YL1, TO1000, and BK2019) and the transgenic plants. The whole genomes of YL1 and BK2019 were sequenced using next-generation sequencing on an Illumina HiSeq 2500 platform (Illumina, San Diego, CA). After analysis with Cutadapt [43] (v1.9.1) and BWA [44] (v0.7.12), the sequencing data for the two parents were aligned to the TO1000 reference genome (http://plants.ensembl.org/Brassica_oleracea) [27], revealing the SNP and InDel variations between BK2019, YL1, and TO1000. Finally, 110 InDel primers were designed based on the following principles: a GC content ranging from 40% to 50%, a midpoint melting temperature (Tm) value ranging from 50°C to60°C, and an amplicon length ranging from 100 to 200 bp. The primers used in this study were synthesized by the Beijing Genomics Institute (Beijing, China), and the sequences of all the primers used are shown in Supplementary Table S1.

### Linkage analyses

The Pur pool was composed of 10 purple F_2_ individuals, and the Gre pool was composed of 10 green F_2_ individuals. Polymorphic markers obtained from YL1 and BK2019 were used to screen the Pur pool and the Gre pool. After the polymorphism of the markers was confirmed, the markers were analyzed using 48 F_2_ individuals constructed from 24 purple individuals and 24 green individuals to confirm whether the markers were linked. Other InDel primers targeted around the linked markers were designed to seek more polymorphic markers. All of the F_2_ individuals were genotyped by the polymorphic markers that were used to construct the genetic linkage map. Polyacrylamide gel electrophoresis and PCR amplification were performed as described by Han [42].

### Sequence analysis of *BoNA1*

Specific primers for *BoNA1* (*BoNA1-F/R*) were designed by Primer3 (https://bioinfo.ut.ee/primer3-0.4.0/) and were used to amplify the full-length gene sequences from YL1, BK2019, the transgenic lines and the other kale varieties. PCR amplification and PCR protocol were performed through the methods described by Yi [28]. The PCR products were then cloned into a pCE2 TA/Blunt-Zero Vector (Vazyme, Nanjing, China). After transformation into FastT1 competent cells, approximately 60 white colonies were randomly picked, and plasmids were extracted from the positive clones by using TIANprep Rapid N96 (TIANGEN Biotech, China). The plasmids were sequenced via the Sanger method by the Beijing Genomics Institute (Beijing, China). Sequence alignment was subsequently performed with DNAMAN version 6.0 software and Chromas software v2.31 (https://www.technelysium.com.au/chromas.html).

The full-length amino acid sequence of BoNA1 was used to search for relatives based on NCBI-based BLASTP searches. Phylogenetic trees were constructed using MEGA7.0 software [45] with the Neighbor-Joining and 1000 replicated bootstrap.

### RNA extraction and gene expression analysis

Using a pure-RNA kit (Vazyme), total RNA was extracted from the hypocotyls of 12-day-old YL1 and BK2019 seedlings; RNA was also extracted from the different tissues of YL1. A cDNA synthesis kit (Vazyme) in conjunction with 1 μg of total RNA was used for reverse transcription. The internal reference gene was *BolActin* (GenBank: AF044573.1) [46, 47]. qRT-PCR amplification and qRT-PCR amplification were preformed according to the methods described by Ji [48]. A CFX96 Real-Time System (Bio-Rad, Hercules, CA) was used to carry out the experiment, and the transcript levels were calculated according to the 2^−ΔΔct^ method [49].

### Knockout vector construction and cabbage transformation

For CRISPR/Cas9 vector construction, we selected one sgRNA targeting the fourth exon of *BoNA1* using the CRISPR-P v2.0 tool (http://cbi.hzau.edu.cn/CRISPR2/). After the fragment was cloned and purified, it was cloned into zmplcas9 (modified from PC1300) via T4 DNA ligase. After DNA sequencing, the vector was introduced by a freeze–thawing process into *Agrobacterium* strain GV3101, which was then used for cabbage transformation. YL1 was used as the transformation receptor, and the method described by Yi for *Agrobacterium-*mediated genetic transformation in *B. oleracea* was used [28].

## Supplementary Material

Web_Material_uhad133

## Data Availability

All the data supporting this work are presented in the manuscript and its supplementary files.
