# Supplementary material for: Map-based cloning and CRISPR/Cas9-based editing uncover BoNA1 as the causal gene for the no-anthocyanin-accumulation phenotype in curly kale (Brassica oleracea var. sabellica)
Source: Hortic Res. 2023 Jun 29;10(8):uhad133. doi: 10.1093/hr/uhad133 (PMC10410298; doi:10.1093/hr/uhad133)
Supplement: Web_Material_uhad133 [file web_material_uhad133.zip › ! Revised Supplementary materials-5.4.docx]

**
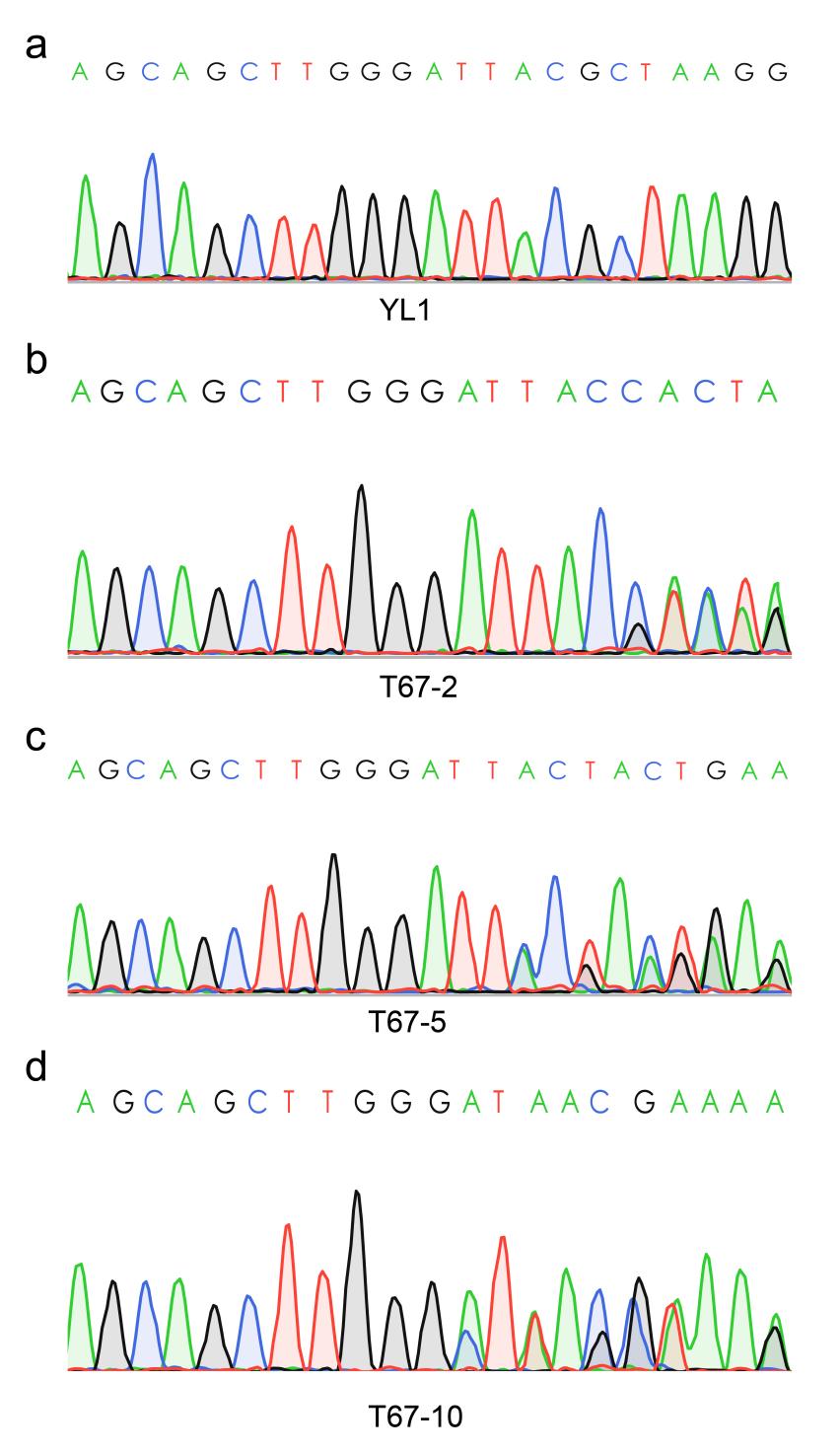
Fig. S1** sgRNA sequencing results of *BoNA1* in YL1, T67-2, T67-5 and T67-10. The red rectangle represents the protospacer-adjacent motif (PAM) sequence.

**Fig. S2** Phenotypes of YL1, 22Q2592 and 22Q2602.


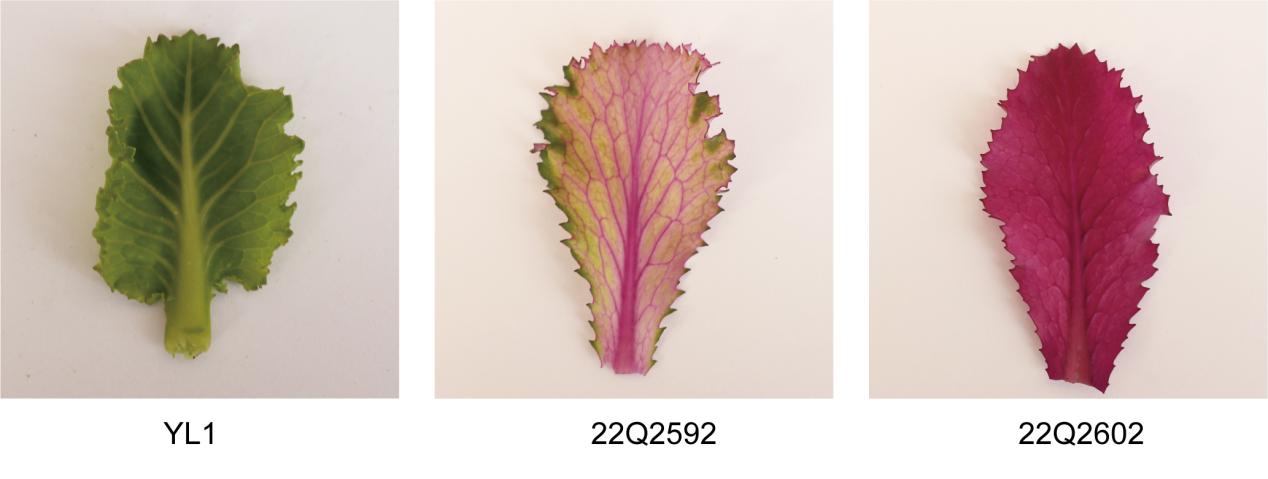


**Fig. S3** Promoter sequence alignment of *BoNA1* in YL1, 22Q2592, and 22Q2602. The yellow lines indicate InDels, and the red rectangle indicates the AE-box.


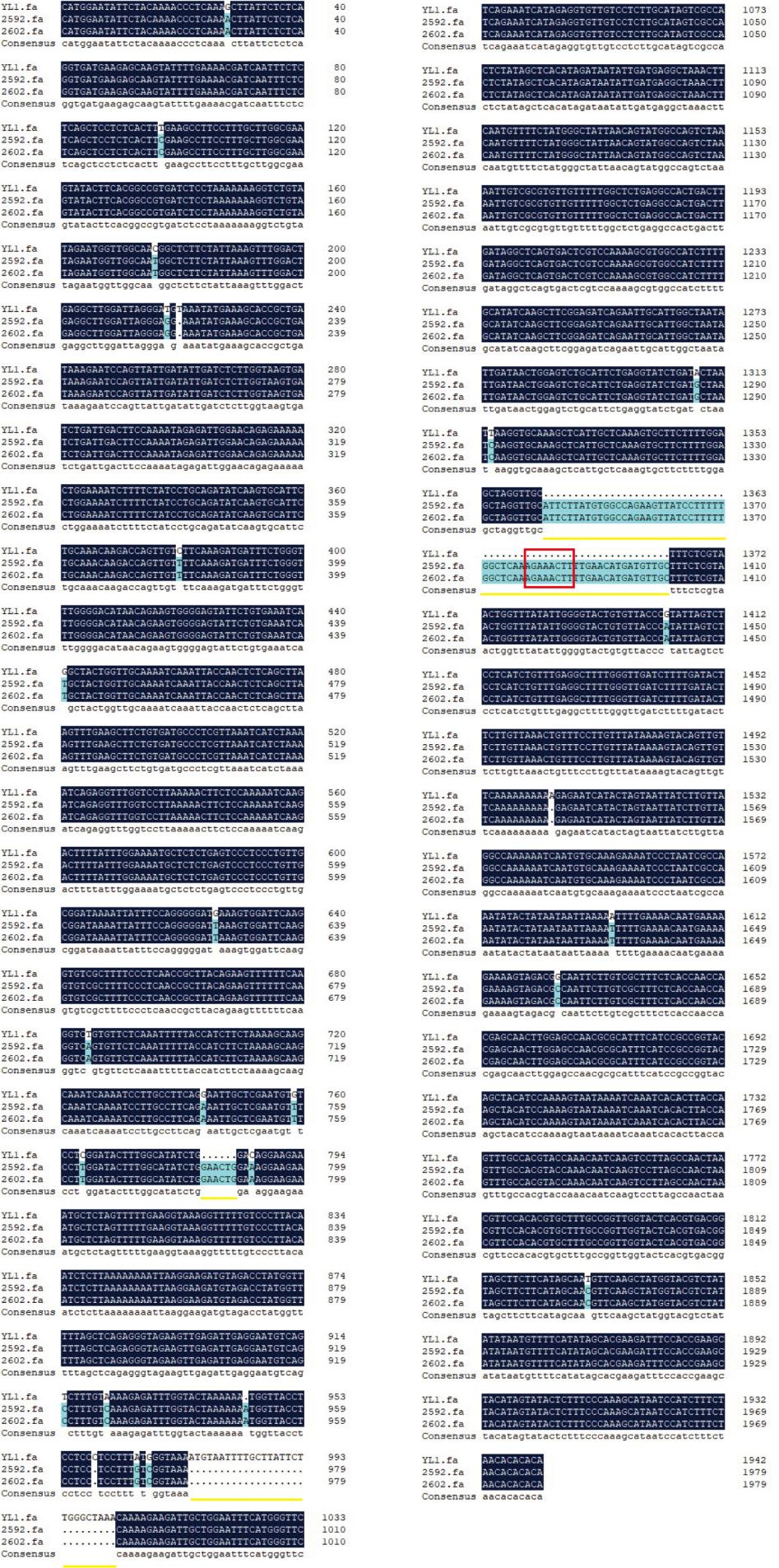
**Fig. S4** Expression of *BoNA1* in YL1, 22Q2592, and 22Q2602.


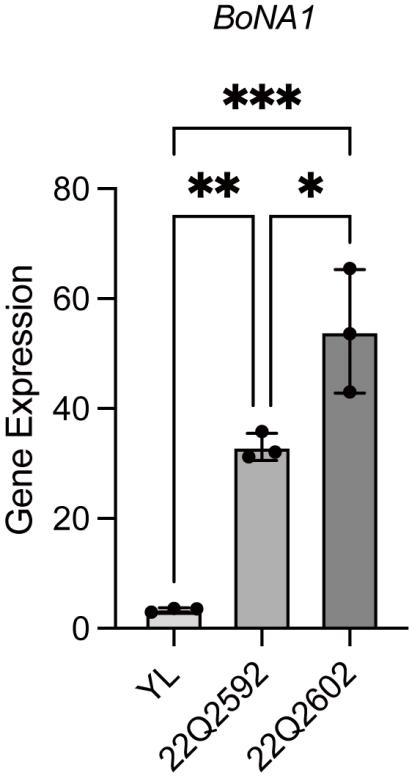


**Table S1** Primer sequences used in this study.

| Primer name | Forward primers sequence (5'-3') | Reverse primers sequence (3'-5') |
| --- | --- | --- |
| P310 | ACCGAGTTCTTGGAATCGTGT | GGTGTTCTCGTACCATGCCT |
| P314 | CAAGGTTCGTCGTCATCCCA | TTCCGCAGCGACATTCCTTT |
| P335 | CCAGTTTGGTACGGTTTGGG | TGCAAGCGCATCCATCTACT |
| P336 | TCCATTCTCCAACGCACACA | ACAGAGGAAACAAAAACCTGT |
| P359 | CCCTGACATCGTAAGCTGCA | TGCAAGGCAGTGTTAGAGAGG |
| P380 | AACTTGCATAAAATAAACTTGGAAAT | GAACAGTTGCAAGGTTAAATCA |
| P382 | GTCACTAGGGACAGGCATCC | ACGTGAAAAATGTCGTGGTGA |
| P523 | CTGAGCCTCGTCCCGGTT | ACTTTCTTTCTTGCTCCTCAGG |
| P531 | CGTTTACAAGGGGAGGCGTA | TCGATCTTTCAATAGACACCTTTCA |
| P536 | AACAAACAATAGACACATAGTATAACA | TGCTAACTAACCGAGTTCACA |
| P539 | CCGGTCAATTTGGCGGAATC | ACAGATTCGTAACCCAACAGCT |
| Cas9 | GACAAGAAGTACTCGATCGGC | GTCAGATCCTGATGGTGCTC |
| Bar | ATGAGCCCAGAACGACGCCCG | TCAAATCTCGGTGACGGGCAGG |
| gBoNA1 | ATAATCCATCTTTCTAACAC | AAATAAGTCCACAACTCTTT |
| qBoNA1 | CCTGGAAATTTGAAGAAAGTGCA | CATCCGTTTATGGCGTCATCGTA |
| BolActin | CCAGAGGTCTTGTTCCAGCCATC | GTTCCACCACTGAGCACAATGTTAC |
| BoNA1-CR | CAGTGGTCTCAAGTGAACAAAGCACCAGTGGTCTA | GTGGTCTCAAAACTAGCGTAATCCCAAGCTGCT |
